# Effect of *Solanum melongena* on Components and Kidney Damage of Fructose-Induced Metabolic Syndrome in Rats

**DOI:** 10.5812/ijpr-139144

**Published:** 2024-01-29

**Authors:** Elizabeth Guzmán Hernández, Maria del Rosario González Valle, José Carmelo Benítez Flores, Maria Eugenia Garian Aguilar, Rubén San Miguel Chávez, Maria Dolores Hernández Martínez, Leonardo del Valle Mondragón, David Segura Cobos, Gil Alfonso Magos Guerrero, Pedro López Sánchez

**Affiliations:** 1 Faculty of Higher Studies Iztacala, National Autonomous University of Mexico, Tlalnepantla, State of Mexico, C.P. 54090, Mexico; 2 Histology Laboratory, Morphology and Function Unit, Faculty of Superior Studies Iztacala, National Autonomous University of Mexico, Tlalnepantla, State of Mexico, 54090, Mexico; 3 Montecillo Campus, Postgraduate College, Texcoco, State of Mexico, Mexico; 4 Department of Pharmacology, Ignacio Chavez National Institute of Cardiology, Mexico City, C.P. 04510, Mexico; 5 Department of Pharmacology, Faculty of Medicine, National Autonomous University of Mexico, Coyoacán, Mexico City, C.P. 04510, Mexico; 6 Postgraduate Studies and Research Section, Higher School of Medicine, National Polytechnic Institute, Mexico, Mexico City, 11340, Mexico

**Keywords:** *Solanum melongena*, Kidney Damage, AT1 Receptor, Cytokine TGF-β1

## Abstract

In traditional Mexican medicine, *Solanum melongena* is used to treat obesity, diabetes mellitus, hypertension, asthma, bronchitis, arthritis, and hypercholesterolemia. This study examined the effect of treatment with ethanolic extract (EE) from the fruit of *S. melongena* on kidney damage on metabolic syndrome (MS). Male Wistar rats were maintained for 12 weeks on a diet of 20% fructose in drinking water and chow to develop MS. After administering EE of *S. melongena* (100 and 200 mg/kg/day, orally) for 6 weeks, the histological study of the kidney cortex and determination by Western blot of the renal expression of angiotensin II AT1 receptor and cytokine transforming growth factor beta 1 (TGF-β1) were realized. The rats fed with high fructose developed MS and showed kidney damage characterized by proliferative glomerulonephritis and necrosis; this damage was reduced by *S. melongena* treatment and associated with decreased expression of AT1 receptor for angiotensin II and cytokine TGF-β1, controlling renal damage in this animal model. In rats affected by MS through high fructose feeding, the treatment with EE of *S. melongena* showed a renoprotective effect through decreased expression of AT1 receptor for angiotensin II and cytokine TGF-β1, causes of glomerulonephritis.

## 1. Background

Overweight and obesity constitute one of the main public health problems in Mexico and are associated with type 2 diabetes mellitus, arterial hypertension, and dyslipidemia, which together make up metabolic syndrome (MS). According to the latest National Health and Nutrition Survey 2021, obesity affects more individuals between 40 and 49 years of age, especially in urban areas; however, overweight is more common in rural areas due to a sedentary lifestyle, increased consumption of processed foods, and sugary drinks, which places Mexico in the second place worldwide regarding obesity (1).

Obesity is considered one of the main factors that predispose patients to the development of kidney damage secondary to diabetes mellitus or high blood pressure; nevertheless, it also produces kidney damage directly through hemodynamic alterations, low-grade inflammatory process, intensity, and deregulation of growth factors and adipokines (2, 3). Growing evidence shows that during the development of MS, there is a deregulation in the activity of the renin-angiotensin system (RAS) associated with kidney damage (4, 5). Recent studies have shown that angiotensin II (Ang II) in proximal tubular cells directly stimulates the transforming growth factor beta 1 (TGF-β1) protein expression, which triggers kidney damage characterized by renal hypertrophy, tubular atrophy, interstitial fibrosis (4). Despite effective drugs for treating these risk factors, in the vast majority of cases, side effects occur in patients, leading them to abandon their treatment and resort to other alternatives, such as traditional medicine.

In Mexico, this knowledge continues to be preserved through the different indigenous. *Solanum melongena* (eggplant), given the agrochemical characteristics of the fruit, the possibilities of preparing a variety of dishes for edible purposes are minimal since it contains alkaloids that are harmful to human health, such as solanine, which is why it can only be eaten cooked as it decomposes due to heat (3).

Recent studies have shown that chlorogenic acid (5-O-caffeoylquinic acid) is the predominant compound in the fruit, followed by 3,5- and 4,5-dicaffeoylquinic acids, caffeic acid, and esters of 3- 5-O- and 4-O-caffeoylquinic O-acetyl (5). The presence of these secondary metabolites helps lower blood cholesterol and triacylglyceride levels in mice and humans by modulating their metabolism and increasing excretion itself. In addition to the aforementioned properties and the high nutritional value, analgesic, anti-inflammatory, and anti-allergic activities have been found (3, 4, 6).

## 2. Objectives

The objective of the present study was to determine the effect of ethanolic extract (EE) of *S. melongena* on MS, its components, and kidney damage in rats.

## 3. Methods

### 3.1. Preparation and Identification of the Ethanolic Extract of Solanum melongena (EE)

In this study, 10 l of ethanol (J.T. Baker) was added to 5 kg of shelled fresh fruit of *S. melongena* and macerated for 48 hours. Extracts were concentrated in a rotary evaporator (Buchi rotavapor model Mp60), and to evaporate the solvent, it was left to dry in an oven at 60°C for 24 hours.

### 3.2. Phytochemical Profiling

For the analysis of the secondary metabolites present in the EE of *S. melongena*, they were determined using a Hewlett Packard Mod. 1100 high-resolution liquid chromatograph equipped with an automatic injector (Agilent Technologies Mod. 1200), a diode detector (Hewlett Packard Mod. 1100), and an HP Mod. 1100 quaternary pump. Phenolic acids were detected at 280 nm and flavonoids at a wavelength of 254, 316, and 365 nm under the following conditions: 

A gradient of 1 mL/min of water (pH 2.5 with trifluoroacetic acid) was used (solution A) in addition to acetonitrile (solution B) (initially, (0 to 0.1 min) 85% solution A and 15% solution B, (0.1 to 20 min) 65% solution A and 35% solution B, and (20 to 23 min) solution A at 65% and solution B at 35%; injection volume: 20 μL). Terpenoids were detected at wavelengths of 215 and 220 nm; the gradient of the mobile phase was 20% water and 80% acetonitrile.

### 3.3. Ethical Considerations and Animals Used 

This study was carried out under the guidelines of the standard Official Mexican Rule (NOM-062-ZOO-1999, revised in 2001). Experimental protocols complied with the European Communities Council Directive of 24 November 1986 (86/609/EEC) and were approved under the Ethics Committee of the Faculty of Superior Studies Iztacala, UNAM, Mexico. 

### 3.4. Induction of Metabolic Syndrome

The control diet (2018s Teklad Global 18% protein rodent diet from Harlan Laboratories) contained proteins (18.6%), carbohydrates (44.2%), and fat (6.2%). Chow and drinking water with 20% fructose were elaborated (7). The rats were initially divided into two groups: The control group (n = 6) and the fructose-fed (F) group (n = 24).

### 3.5. Experimental Design

After 12 weeks of fructose treatment, the rats were randomly divided into four groups (n = 6) and were maintained under initial diet conditions. The treatments were orally administrated for 6 weeks in the following groups: (1) Control group; and (2) MS.

**Figure 1.**
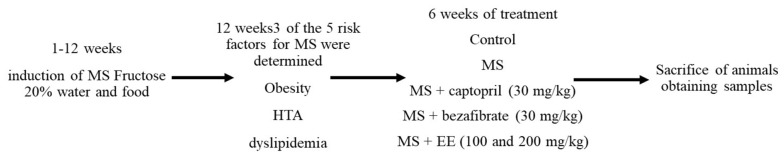
Timeline of the experimental phase

- Metabolic syndrome treated with captopril 30 mg/kg (MS + CAP): Studies carried out (6, 8) demonstrated that the administration of 30 mg/kg of captopril has a cardiovascular effect since by inhibiting the activity of angiotensin converser enzyme (ACE), bradykinin is not degraded and can exert its cardiovascular effects through the synthesis of prostanoids, such as prostacyclin.

- The MS group treated with bezafibrate 10 mg/kg [MS + bezafibrate (BEZA)]: For its cardioprotective effects through the decrease in the availability and absorption of systemic fatty acids by the muscle was used bezafibrate (8).

- Metabolic syndrome treated with the EE of *S. melongena* (EE): 100 mg/kg and 200 mg/kg (MS + EE 100; MS + EE 200) (Figure 1). 

After 6 weeks of treatment, the animals were placed in metabolic boxes for 24 hours for the determination of water consumption, food, urinary volume, and urine sample collection.

### 3.6. Obesity Parameters

The percentage of body weight gain, Body Mass Index (BMI), abdominal circumference (AC), and Lee index were measured as indicators of obesity. Body weight was taken every week using a weighing scale. The increment of body weight was calculated by subtracting the final weight.

 The Lee index was calculated by the cube root of body weight (g) × 10/naso-anal length (mm). The BMI was quantified by dividing the weight (g) by the length (cm^2^) (9). The length of the rats was measured between nasal and anal region (10). The abdominal circumference was measured with tape around the anterior abdomen in cm (9). 

### 3.7. Blood Pressure

Systolic arterial blood pressure (SBP) was measured noninvasively using a tail cuff (8).

### 3.8. Biochemical Analyses

To determine the presence of proteins in the urine, the Bradford method was used (11).

The determination of glucose, cholesterol, and triglycerides (TG) was carried out using an Accutrend Sensor glucometer (Roche). At the end of the treatment, 3 mL of venous blood was taken for the determination of high-density lipoprotein cholesterol (HDL-C) and low-density lipoprotein cholesterol (LDL-C) was measured by commercially available kits (Spinreact) (7). Through capillary zone electrophoresis, the plasma concentration of angiotensin II, angiotensin (1-12, 8, 13), and nitric oxide (13). For the determination of very-low-density lipoprotein cholesterol (VLDLc), atherogenic index and coronary artery index were calculated according to what was described by (11).

### 3.9. Histology and Western Blotting

The kidneys were perfused and rapidly removed. The right kidney was used for histological analysis; therefore, it was fixed in 4% formaldehyde. Subsequently, it was dehydrated with alcohol at different concentrations until it was mounted and stained with hematoxylin-eosin; however, the left kidney was used for immunoblot. 

The renal cortex of the left kidney was separated by 10% (w/v) polyacrylamide gel electrophoresis and electroblotted to polyvinylidene difluoride membranes. Membranes were blocked for 2 hours in 5% (w/v) nonfat milk and incubated overnight in the presence of antibodies (rabbit polyclonal antibody to AT1 receptor (AT1R), mouse monoclonal antibodies to TGF-β1 and β-actin) (1: 1000 dilution) and for 2 hours at room temperature in the presence of horseradish-peroxidase-conjugated secondary antibodies (1: 1000 dilution). All antibodies used were obtained from Santa Cruz Biotechnology Inc. (Santa Cruz, California, USA). Complexes were visualized by chemiluminescence detection. Films were scanned, and densitometric analysis was carried out using Multi Gauge software (version 2.1), Fujifilm Science, Lab 2003, Fuji Photo Film Co., Tokyo, Japan) (14).

### 3.10. Statistical Analysis

The data were analyzed through a two-factor analysis of variance (ANOVA) in [GraphPad software (version 5.0); La Jolla, California, USA], and the interactions were analyzed by Tukey’s multiple comparison post hoc test. 

## 4. Results

### 4.1. Identification of Phenolic and Flavonoid Compounds in Ethanolic Extract of Solanum melongena

**Figure 2.**
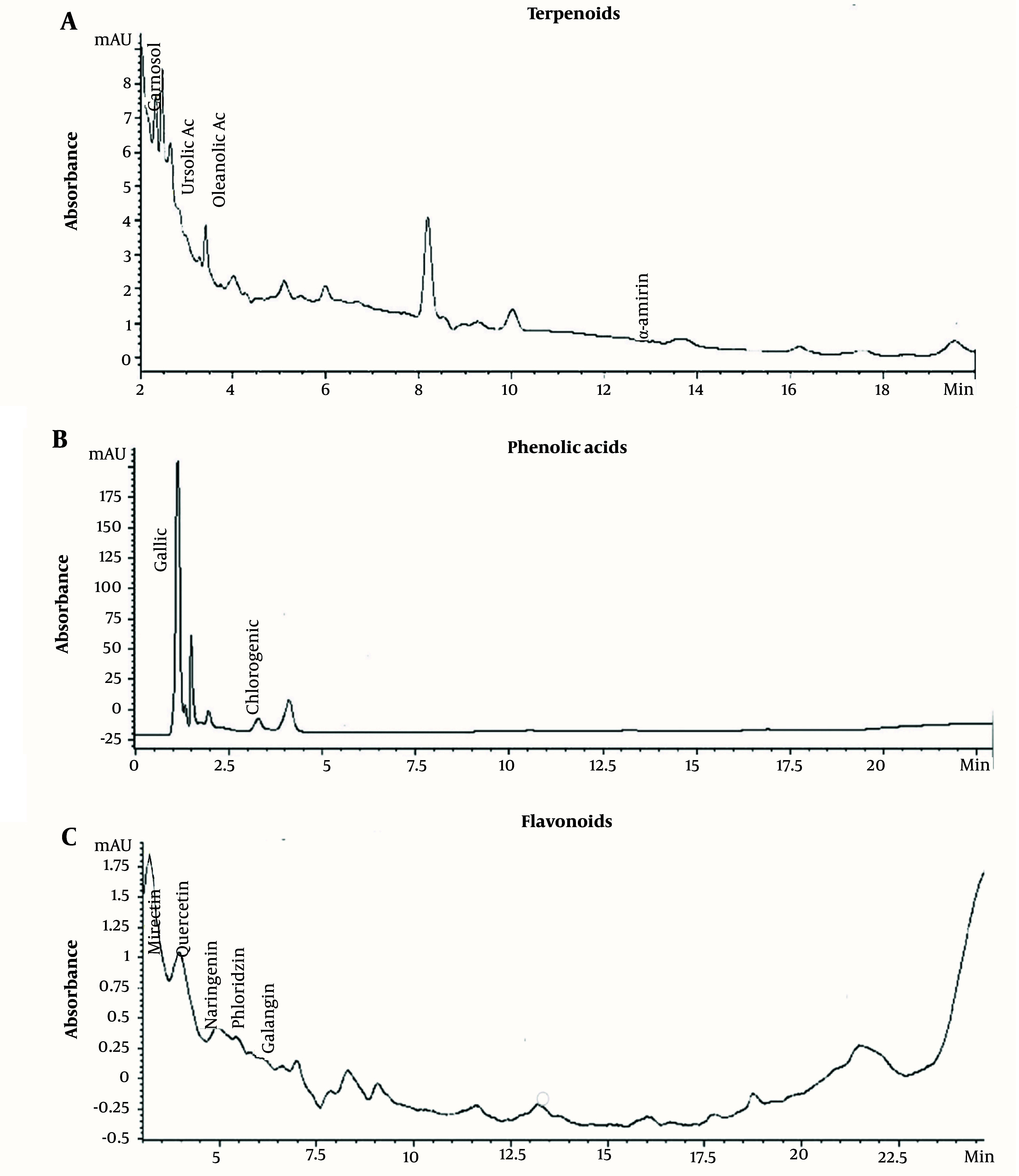
**C**hromatographic profile of terpenoids, phenolic acids, and flavonoids of ethanolic extract of *Solanum melongena*. Among the main compounds found are -amyrin (13.47%), phloretin (3.15%), chlorogenic acid (2.7%), galangin (2.44%), and phloridzin (2.31%).

Table 1 and Figure 2 show the most abundant phytoconstituents found in the EE of *S. melongena*, α-amyrin (13.47%), phloretin (3.15%), galangin (2.44%), phloridzin (2.31%), chlorogenic acid (2.7%), ursolic acid (1.55%), oleanolic acid (2%), and naringenin (1.74 %). 

**Table 1.** Phytochemicals Detected in the Ethanolic Extract of *Solanum melongena*, with the Major Presence of Alpha Amyrin

Retention Time (min)	Area (mAU*s)	Metabolite	Amount (μg)
**12.404**	61.0620	Naringenin	1.74
**21.462**	704.365	Galangin	2.44
**6.594**	171.133	Phloridzin	2.31
**7.838**	157.0467	Mirecetin	0.95
**10.938**	79.9739	Quercetin	1.09
**13.179**	361.465	Floretin	3.15
**6.192**	51.674	α-amyrin	13.47
**4.149**	139.690	Oleanolic acid	2
**2.651**	94.9141	Ursolic acid	1.55
**4.088**	487.4322	Chlorogenic acid	2.7
**1.732**	96.4534	Gallic acid	0.072

### 4.2. Effect of Ethanolic Extract of Solanum melongena on Metabolic Syndrome

**Figure 3.**
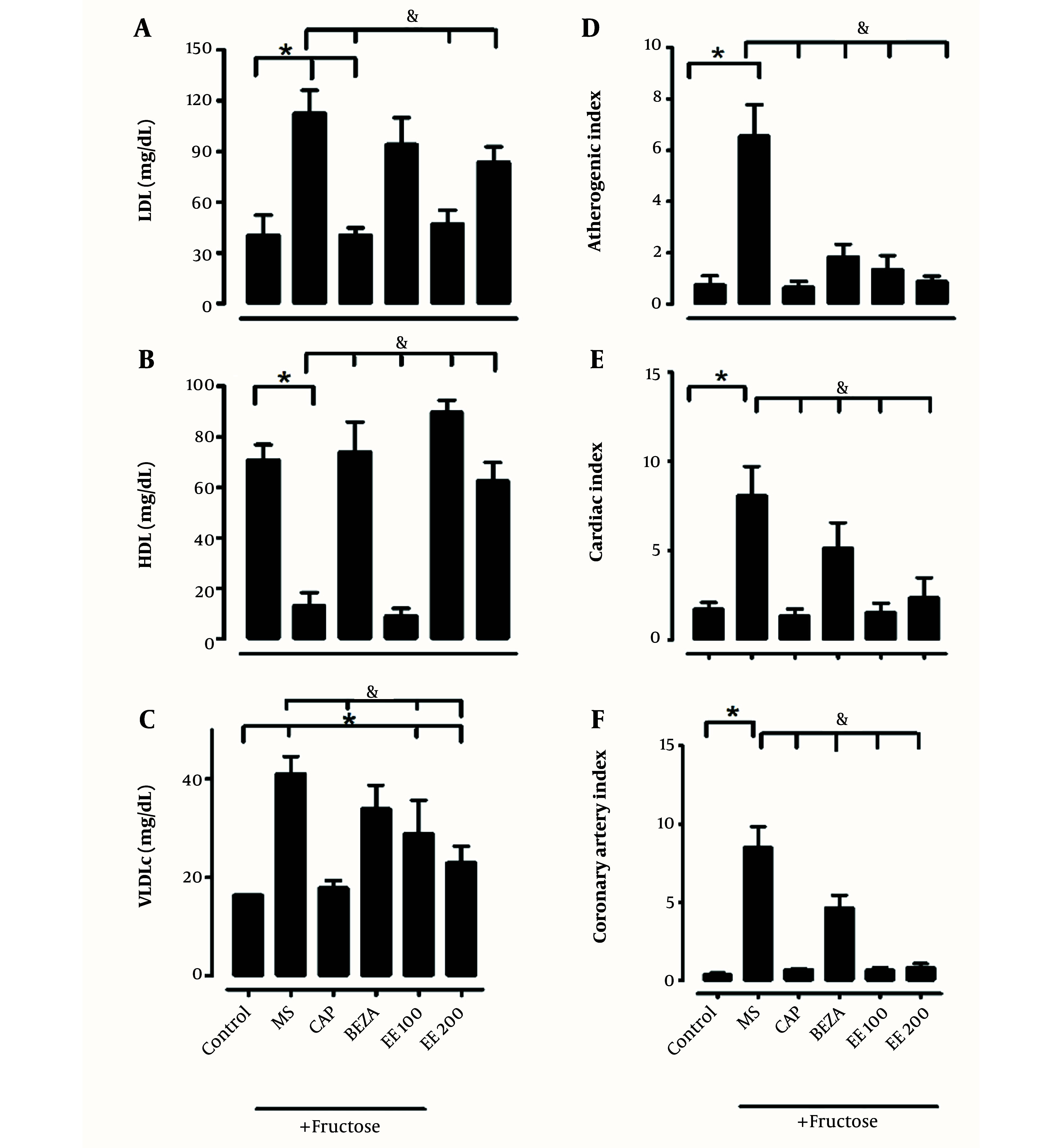
Lipid profile in the presence of the ethanolic extract of *Solanum melongena* [*: Control vs. treatment; &: Metabolic syndrome (MS) vs. treatment: MS + captopril (CAP), MS + bezafibrate (BEZA), MS + ethanolic extract of *S. melongena* (EE 100 and MS + EE 200 mg/kg)]. The lipid profile altered by MS tended to be normalized by EE treatment.

Twelve weeks after the induction of MS, the animals developed 3 of 5 risk factors: Arterial hypertension (118 ± 0.69 vs. 150 ± 3 mmHg, P < 0.05), dyslipidemia (0.91 ± 0.25 vs. 1.91 ± 0.27 mmol/L, P < 0.05), and obesity. In the presence of treatments captopril, bezafibrate, and EE, obesity indicators improved (Table 2). The atherogenic, coronary, and cardiac indexes, LDL-C, and TG increased in the group with MS without treatment (Figure 3), and administration of EE (100 and 200 mg/kg), has ameliorated lipid profile and cardiovascular risk indicators.

**Table 2.** Effect of Ethanolic Extract Treatment on Metabolic Variables in Metabolic Syndrome ^a, b^

Variables	Control	MS	CAP	BEZA	EE 100	EE 200
**Body weight (g)**	490 ± 20	572 ± 19 ^c^	468 ± 16 ^d^	575 ± 29	548 ± 26	529 ± 24 ^c, d^
**Visceral Adiposity Index (%)**	3 ± 0.4	6 ± 0.5 ^c^	4 ± 0.5 ^d^	5 ± 0.4 ^d^	4 ± 0.3 ^d^	4 ± 0.30 ^d^
**Body Mass Index (kg/m** ^**2**^ **)**	0.73 ± 0.02	1.05 ± 0.11 ^c^	0.76 ± 0.03 ^d^	0.74 ± 0.05 ^d^	0.75± 002 ^d^	0.77 ± 0.02 ^d^
**Retroperitoneal**	177 ± 20	335 ± 12 ^b^	185 ± 20 ^c^	220 ± 24 ^c^	271 ± 12 ^c^	260 ± 20 ^c^
**Adipose tissue omental**	170 ± 24	188 ± 9	191 ± 29	221 ± 7	162 ± 28	173 ± 35
**Plasma glucose (mmol/L)**	81 ± 5	97 ± 5 ^c^	87 ± 7	109 ± 10	99 ± 8	114 ± 4
**Plasma triglycerides (mmol/L)**	80 ± 3	224 ± 10 ^c^	88 ± 8 ^d^	171 ± 25 ^c, d^	174 ± 12 ^c, d^	116 ± 18 ^c, d^
**Plasma cholesterol (mmol/L)**	80 ± 2	137 ± 15 ^c^	100 ± 2 ^c, d^	100 ± 2 ^c, d^	100 ± 2 ^c, d^	100 ± 2 ^c, d^

^a^ Control, syndrome (MS), MS + captopril (CAP), MS + bezafibrate (BEZA), MS + ethanolic extract (EE) (100 and 200 mg/kg).

^b^ In rats with MS, a 17% increase in body weight, a 44% increase in Body Mass Index, and a doubling of Visceral Adiposity Index were observed.

^c^ Control vs. treatment.

^d^ Metabolic syndrome vs. treatment.

### 4.3. Antihypertensive Effect of Ethanolic Extract

**Figure 4.**
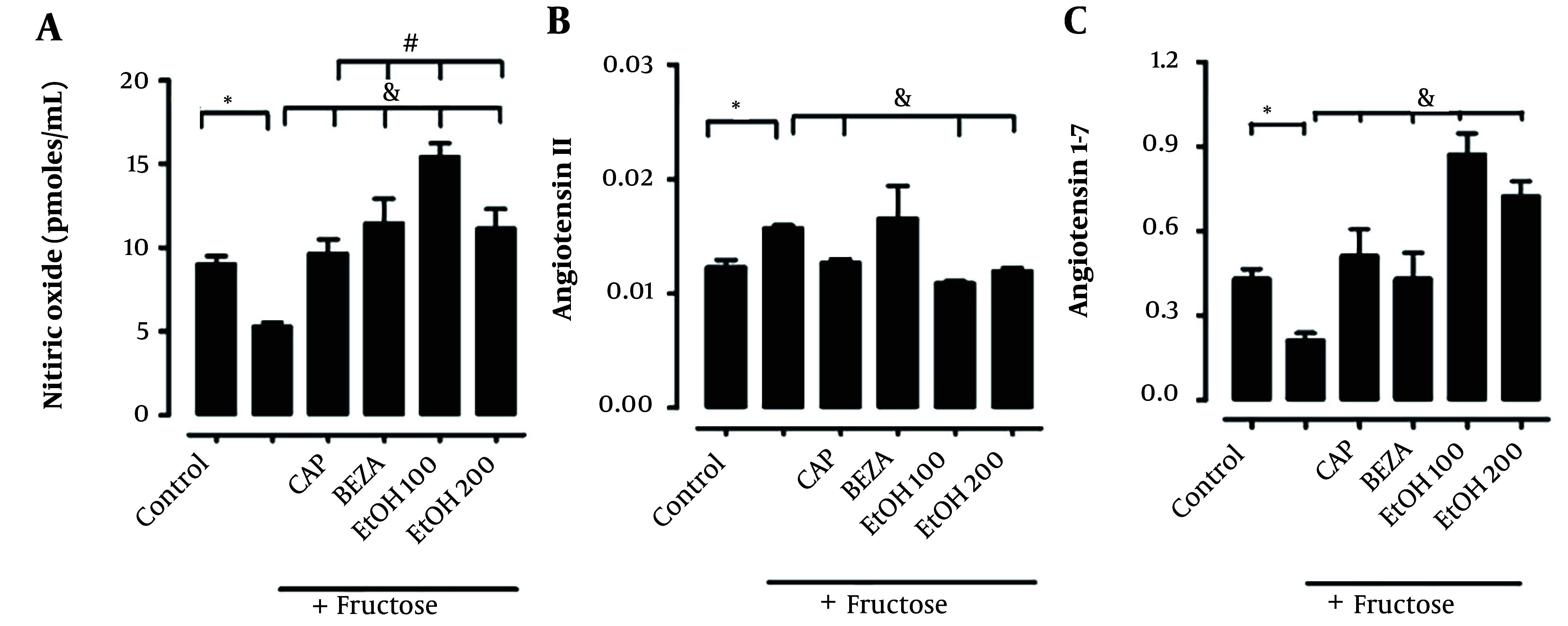
Plasma level of A, nitric oxide; B, angiotensin II; and C, angiotensin 1-7 in the presence of ethanolic extract of *Solanum melongena* [*: Control vs. treatment; &: Metabolic syndrome (MS) vs. treatment; MS + captopril (CAP); MS + bezafibrate (BEZA), MS + ethanolic extract of *S. melongena* (EE 100 and MS + EE 200 mg/kg)].

The systolic blood pressure was increased (150 ± 3 mmHg in MS group compared to 100 ± 2 mmHg in the control group, P < 0.05); MS + captopril group (110 ± 3 mmHg) and MS + EE 100 and 200 mg/kg (113 ± 5 and 110 ± 4 mmHg). To determine the possible mechanism of action of the antihypertensive effect of EE from *S. melongena*, in vitro experiments were carried out in aortic rings with and without endothelium. In the presence of vascular endothelium, the incubation with EE of *S. melongena* in concentrations of 0.03 to 3 (ng/mL) has caused a 60% relaxation. In the absence of endothelium, this relaxation percentage is reduced, which suggests that the antihypertensive effect observed *in vivo* is dependent on vascular endothelium and nitric oxide liberation. When were quantitied the vasoactive peptides using zone capillary electrophoresis, was observed an increased plasma Ang II. Ang (1 - 7) and nitric oxide were slightly decreased in the MS group (Figure 4A), and EE of *S. melongena* (100 and 200 mg/kg) has caused a significant decrease in plasma Ang II and has increased plasma Ang 1-7 and nitric oxide (Figure 4B and C), which suggests that the antihypertensive effect of EE of *S. melongena* is through nitric oxide and Ang 1-7 (Figure 4B and C). 

As an indicator of kidney function, the presence of proteins in urine in MS increased significantly as compared to the control group (C: 24 ± 3, F: 60 ± 4 mg/24 h, P < 0.05); the treatment with *S. melongena* 100 and 200 mg/kg reversed this increment (25 ± 3 and 20 ± 3 mg/24 h, P < 0.05).

**Figure 5.**
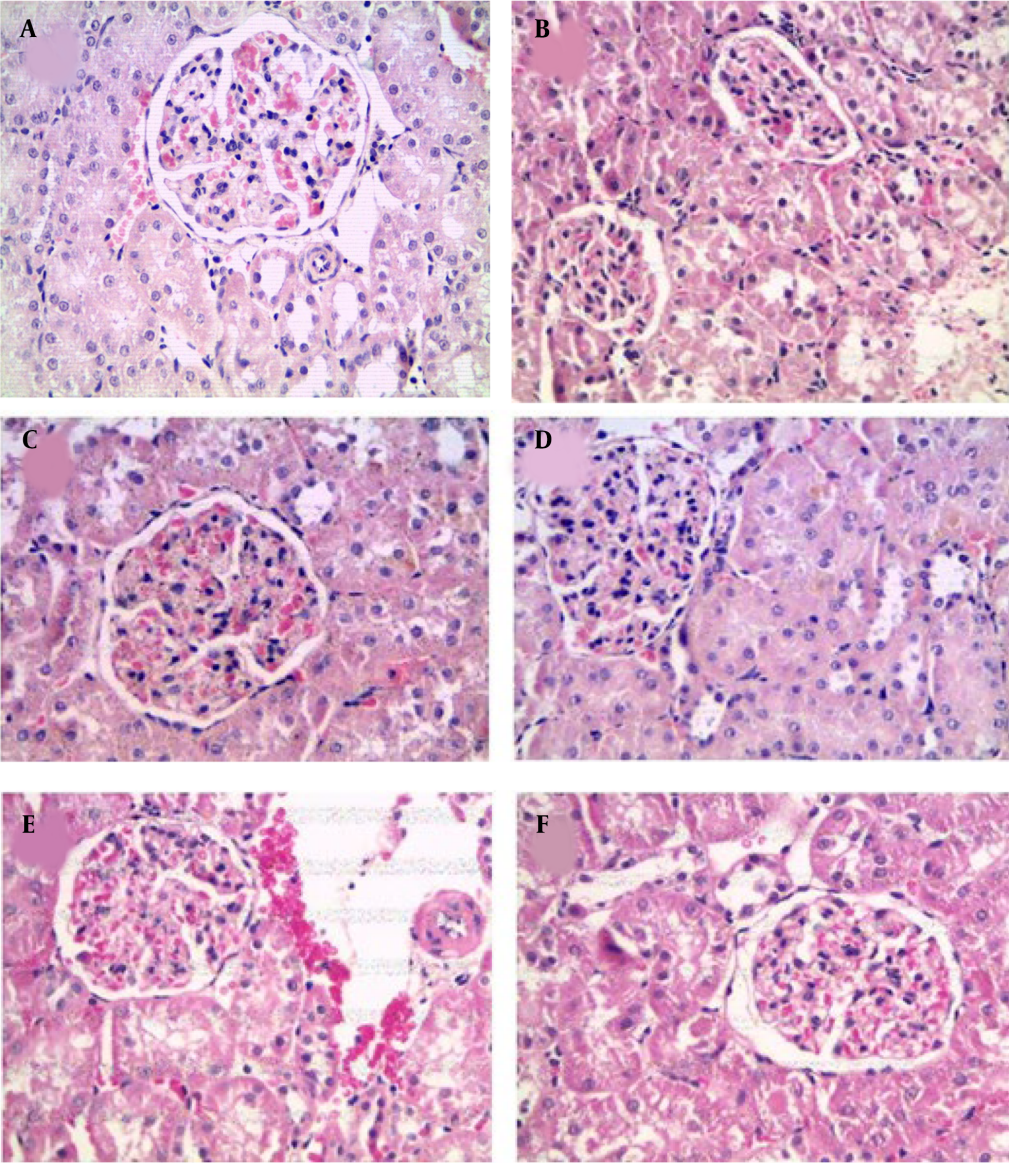
Photomicrography of histological sections of the kidney. A, control; B, metabolic syndrome; C, captopril; D, bezafibrate; E and F, ethanolic extract of fruit of *Solanum melongena* (100 and 200 mg/kg)

The histological analysis of MS showed kidney glomerulonephritis multifocal. Atrophy and necrosis were observed in the proximal and distal tubules (Figure 5B). 

All these derangements observed in fructose-fed rats were partially ameliorated by treatment with captopril and bezafibrate. The main effect is on the restoration of glomerular function; in these treatments, glomeruli are well infused, accompanied by decreased hypercellularity. Renal corpuscles are generally observed to be of normal size and structure (Figure 5C and D). 

In treatments with EE of *S. melongena* (100 and 200 mg/kg), glomeruli were observed with endothelial cells, podocytes, and mesangial cells with hyperchromatic nuclei. Hypercellularity was maintained, and no atrophic corpuscles were observed. The overall appearance is functional corpuscles (Figure 5E and F). In proximal tubules, observing degenerative and necrotic changes decreased significantly, and diffuse areas of tubular regeneration were identified; the latter was valued for the presence of small tubules with low prismatic cells and little development of microvellosity region. The cytoplasmic and nuclear aspects of epithelial cells are normal; these tubules without lumen were considered young (Figure 5E and F).

### 4.4. Effect of Ethanolic Extract of Solanum melongena on Damaged Kidney 

**Figure 6.**
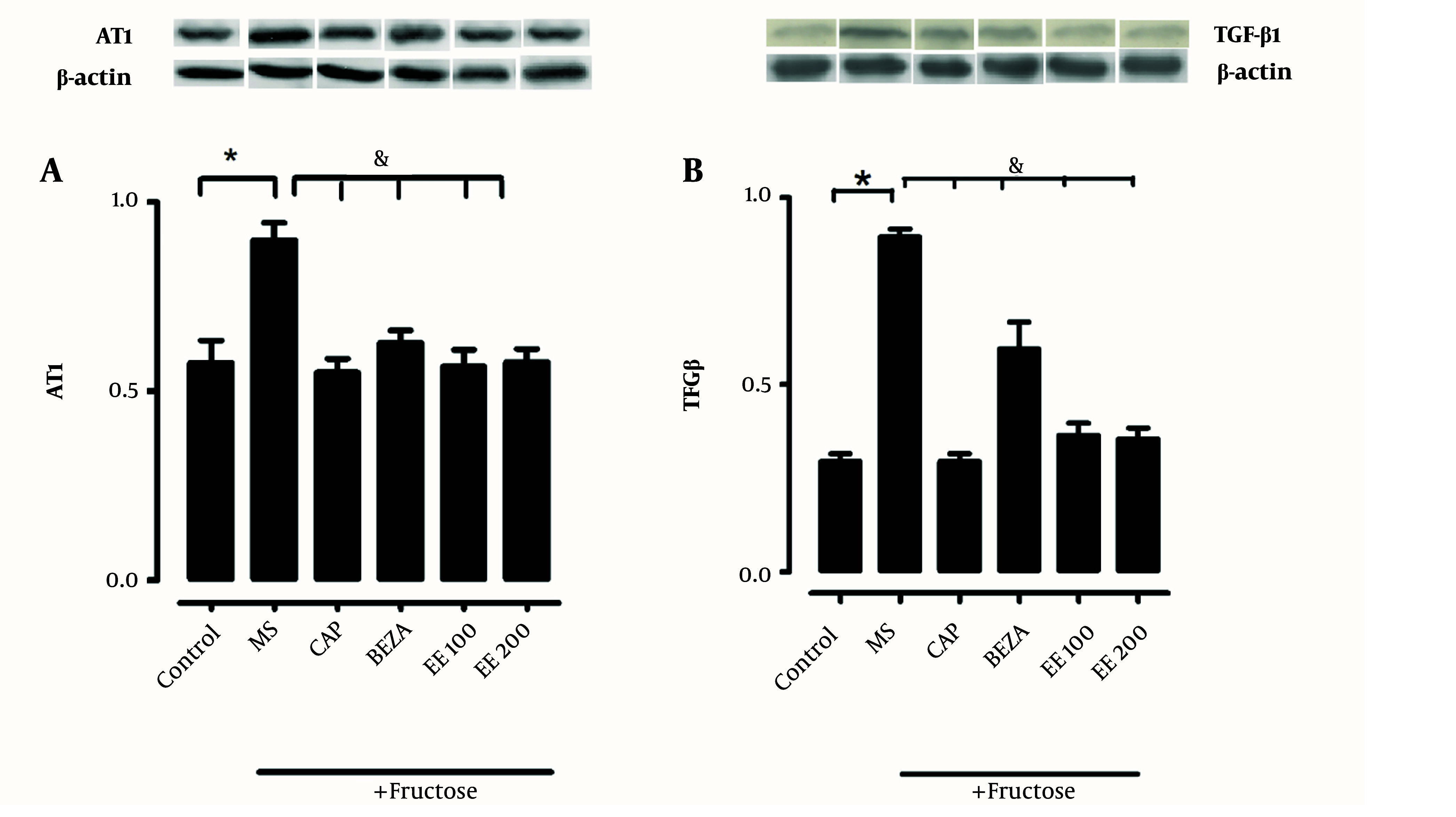
Effects of ethanolic extract of *Solanum melongena* on the expression of A, AT1 receptor (AT1R); and B, transforming growth factor beta 1 (TGF-β1) proteins in renal cortex [*: Control vs. treatment; &: Metabolic syndrome (MS) vs. treatment. MS + captopril (CAP), MS + bezafibrate (BEZA), MS + ethanolic extract (EE) (100 and 200 mg/kg)]. There was an elevated kidney expression of AT1R and TGF-β1 in MS rats compared to the control group (Figure 5A and B). The captopril, bezafibrate, and EE of *S. melongena* (100 and 200 mg/kg) treatments significantly diminished AT1R and TGF-β1 expression.

One of the components of MS is obesity, which is related to the development of kidney damage and is associated with elevated expression of AT1R and TGF-β1 (Figure 6). Another certain indicator was hypertrophy through renal weight/total body weight ratio. The presence of hypertrophy in MS was observed in comparison to the control group (C: 2.0 ± 0.02, F: 3.5 ± 0.02 mg/g, P < 0.05); the treatment with EE of *S. melongena* 100 and 200 mg/kg reversed this increment (2.0 ± 0.18 and 1.8 ± 0.27 mg/g, P < 0.05). This result suggests an antihypertrophic effect of *S. melongena*.

The expression of the AT1 and TGF-β receptors increased in animals with MS (Figure 6A and B). The captopril, bezafibrate, and EE of *S. melongena* (100 and 200 mg/kg) treatment significantly diminished AT1R and TGF-β1 expression (Figure 6A and B).

## 5. Discussion

Fructose-fed rats developed arterial hypertension, dyslipidemia, and visceral obesity, manifesting MS. In this model of MS, the EE of *S. melongena* showed a renoprotective effect through decreased expression of the AT1 receptor and cytokine TGF-β1, which would reduce renal fibrosis and/or inflammation. In early stage of DM in rats, was observed an increased renal cortical AT1 receptor protein and circulating Ang II levels consistent with an exaggerated influence of Ang II on renal function. The increase in AT1 receptor protein expression reflects an increase in functional receptors that are coupled to intracellular pathways; these changes can be expected to augment Ang II-dependent influences on renal function and might also contribute to the growth-promoting processes that engender the eventual development of nephropathy.

According to the low percentage of fructose used in the present study, glucemia was not modified. So, EE of *S. melongena* did not affect blood glucose levels. This effect might be due to the percentage of fructose used, which, according to other studies, corresponds to a low percentage (9, 12). Therefore, the development of factors associated with MS depends on the time of exposure to the fruit and the percentage used. According to the conditions described, it maybe insulin resistance was just developed, and modification in the lipid profile due to an increase in the synthesis of free fatty acids, which are deposited in the abdominal cavity, which begins to establish changes in sensitivity to hormones, such as insulin and leptin that participate in the regulation. The regulation of satiety is modified with time of exposure to fructose, which leads to a gain in body weight, as has been observed in other studies in which percentages of 12.5% fructose have been used for 10 weeks (15).

As indicators of obesity, body weight gain was determined after 18 weeks of treatment through the Lee index, BMI, and AC, which increased in animals treated with fructose, compared to the control group. Previous studies have determined that Lee’s index > 0.30, BMI, and AC > 0.68 cm are considered obesity (9, 14-16). The data obtained from this study are consistent with previous results on high-fructose diet-mediated alterations in these parameters. The treatment with EE of fruit of* S. melongena* in MS rats decreased the indicators of obesity and modified the lipid profile. This effect may be due to the presence of flavonoids, such as naringenin, galangin, myricetin, or alpha-amyrin, which can contribute to inhibition of the synthesis of short-chain fatty acids and delay gastric emptying. Therefore, nutrient reabsorption occurs more slowly (9, 17).

### 5.1. Antihypertensive Effect of Ethanolic Extract

The deposition of free fatty acids on endothelial cells contributes to the loss of the synthesis of vasoactive vasodilator mediators that contribute to the homeostasis of blood pressure regulation. After 12 weeks of induction of the MS, changes in synthesis of vasoactive vasodilator nediators was observed. In MS rats group was observed a decreased systemic synthesis of vasodilator nitric oxide and angiotensin 1-7, and an increase of vasoconstrictor angiotensin II (Figure 4A-C), as has been shown by other authors (9, 17).

In the presence of the EE of *Solanum melongena*, blood pressure was normalized. This effect is due to the presence of flavonoids chlorogenic acid, oleanolic, and ursolic acid, which cause angiotensin-converting enzyme inhibition (18) or block calcium from entering the cell (19-21). 

### 5.2. Kidney Damage 

Obesity is considered one of the determining factors in the appearance of kidney damage, in which multifocal glomerulonephritis can be observed, accompanied by degenerative and necrotic changes (18-20). The treatment with captopril and bezafibrate ameliorates derangements observed in MS, similar to a report in another work (21).

At treatments with EE of *S. melongena* of fructose-fed rats, glomeruli were observed with endothelial cells, podocytes, and mesangial cells with hyperchromatic nuclei, hypercellularity was maintained, and no atrophic corpuscles were observed. Possibly, this effect is due to the presence of oleanolic (22, 23) and ursolic acid (24) quercetin (25) myricetin (26) and galangin (27) which have shown nephroprotective effects.

In humans and animal models, hyperinsulinism causes endothelial dysfunction, characterized by a deficiency in the synthesis of nitric oxide and increased actions of angiotensin II, thereby establishing a direct link between hypertension, obesity, and MS (28, 29). The present study showed the elevated expression of AT1R and TGF-β1 in MS. Other studies showed that Ang II increases the expression of TGF-β1 hepatic cells (29). The captopril (ACE inhibitor), bezafibrate (lipoprotein lipase stimulator), and EE of *S. melongena* diminished Ang II concentrations and AT1R expression. The results of the present study are in agreement with those described by other authors who have shown the highest angiotensin-converting enzyme inhibitory activity (22). 

### 5.3. Conclusions

Fructose-fed rats developed arterial hypertension, dyslipidemia, and visceral obesity, manifesting MS. Ethanolic extract treatment in MS rats prevented the increase of SBP, led to weight loss, and could reverse plasma lipid changes altered by MS, such as TG, TC, HDL‐C, LDL‐C, and LDL‐C/HDL‐C. In rats affected by MS, the treatment with EE of *S. melongena* showed a renoprotective effect through decreased expression of AT1 receptor for angiotensin II and cytokine TGF-β1, causes of glomerulonephritis.

## Data Availability

The dataset presented in the study is available on request from the corresponding author during submission or after publication.
